# Dielectric multi-momentum meta-transformer in the visible

**DOI:** 10.1038/s41467-019-12637-0

**Published:** 2019-10-21

**Authors:** Lei Jin, Yao-Wei Huang, Zhongwei Jin, Robert C. Devlin, Zhaogang Dong, Shengtao Mei, Menghua Jiang, Wei Ting Chen, Zhun Wei, Hong Liu, Jinghua Teng, Aaron Danner, Xiangping Li, Shumin Xiao, Shuang Zhang, Changyuan Yu, Joel K. W. Yang, Federico Capasso, Cheng-Wei Qiu

**Affiliations:** 1 Department of Electrical and Computer Engineering, National University of Singapore, 4 Engineering Drive 3, Singapore, 117583 Singapore; 2 Harvard John A. Paulson School of Engineering and Applied Sciences, Harvard University, Cambridge, MA 02138 USA; 3 Institute of Materials Research and Engineering, A*STAR (Agency for Science, Technology and Research), 2 Fusionopolis Way, #08-03 Innovis, Singapore, 138634 Singapore; 4 Guangdong Provincial Key Laboratory of Optical Fiber Sensing and Communications, Institute of Photonics Technology, Jinan University, Guangzhou, 510632 People’s Republic of China; 5 Ministry of Industry and Information Technology Key Lab of Micro-Nano Optoelectronic Information System, Harbin Institute of Technology, Shenzhen, Guangdong 518055 People’s Republic of China; 6 School of Physics and Astronomy, University of Birmingham, Birmingham, B15 2TT UK; 7 Department of Electronic and Information Engineering, The Hong Kong Polytechnic University, Hung Hom, Kowloon Hong Kong; 8 Singapore University of Technology and Design, 8 Somapah Road, Singapore, 487372 Singapore

**Keywords:** Materials for devices, Nanophotonics and plasmonics, Metamaterials, Sub-wavelength optics

## Abstract

Metasurfaces as artificially nanostructured interfaces hold significant potential for multi-functionality, which may play a pivotal role in the next-generation compact nano-devices. The majority of multi-tasked metasurfaces encode or encrypt multi-information either into the carefully tailored metasurfaces or in pre-set complex incident beam arrays. Here, we propose and demonstrate a multi-momentum transformation metasurface (i.e., meta-transformer), by fully synergizing intrinsic properties of light, e.g., orbital angular momentum (OAM) and linear momentum (LM), with a fixed phase profile imparted by a metasurface. The OAM meta-transformer reconstructs different topologically charged beams into on-axis distinct patterns in the same plane. The LM meta-transformer converts red, green and blue illuminations to the on-axis images of “R”, “G” and “B” as well as vivid color holograms, respectively. Thanks to the infinite states of light-metasurface phase combinations, such ultra-compact meta-transformer has potential in information storage, nanophotonics, optical integration and optical encryption.

## Abstract (web summary)

Here, the authors demonstrate a multi-momentum transformation metasurface. The orbital angular momentum meta-transformer reconstructs different vortex beams into on-axis distinct patterns, and the linear momentum meta-transformer converts red, green and blue beams to vivid color images.

## Introduction

Metasurfaces composed of tailored nanostructures arranged two-dimensionally hold great capabilities to locally control light’s phase, amplitude and polarization states at the subwavelength scale^1–7^. Due to this control capability, metasurfaces can provide specific transmission (T) and reflection (R) functions to work as planar photonic components carrying customized information. Such information can be unlocked via a distinguished field distribution reconstructed at the observed region. When an incident light **U**_**inc**_(*x*_0_, *y*_0_) impinges upon a metasurface ${\mathbf{U}}_{{\mathbf{meta}}}^{{\mathbf{T}}/{\mathbf{R}}}\left( {x_0,y_0} \right)$, the field distribution can be expressed by the convolution of ${\mathbf{U}}_{{\mathbf{meta}}}^{{\mathbf{T}}/{\mathbf{R}}}\left( {x_0,y_0} \right){\mathbf{U}}_{{\mathbf{inc}}}\left( {x_0,y_0} \right)$ and an impulse response *h*(*x*, *y*, *z*) that relates the fields at the metasurface. Therefore, the field distribution on the observation plane can be expressed as (more detail are shown in Supplementary Note 1):

1 $${\mathbf{U}}\left( {x,y,z} \right) = \iint\limits^{\! + \infty }_{\!\!\!-\infty } {{\mathbf{U}}_{{\mathbf{meta}}}^{{\mathbf{T}}/{\mathbf{R}}}} \left( {x_0,y_0} \right){\mathbf{U}}_{{\mathbf{inc}}}\left( {x_0,y_0} \right)h\left( {x - x_0,y - y_0,z} \right)dx_0dy_0$$

The diversity of metasurfaces’ function ${\mathbf{U}}_{{\mathbf{meta}}}^{{\mathbf{T}}/{\mathbf{R}}}\left( {x_0,y_0} \right)$ serves as the base for realizing lenses^8–10^, color^11–14^, polarization filters^15,16^ and holograms^17–20^.

**Fig. 1:**
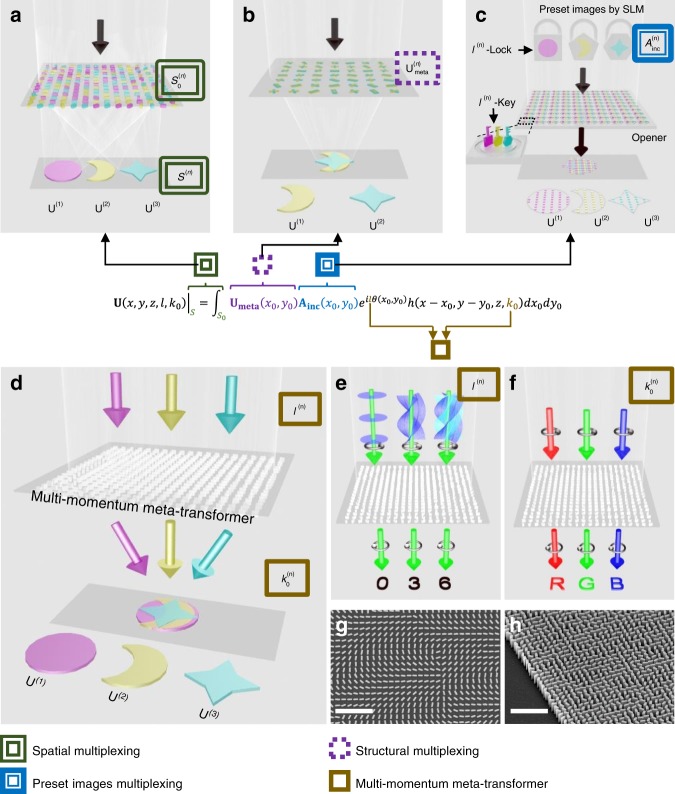
Comparative elaborations of multi-tasked metasurfaces (**a**–**c**) and our multi-momentum meta-transformer (**d**–**h**). **a** Schematic illustration of a spatially multiplexed metasurface^21–29^. The spatial multiplexed metasurface reconstructed several distinguished field distribution ${\mathbf{{U}}}^{({\boldsymbol{n}})}$ based on the spatial separations of metasurface $\left( {S_0^{(n)}} \right)$ or observed region (*S*^(*n*)^). The *x*_0_ and *y*_0_ (*x*, *y*, and *z*) are the coordinated variables of the metasurface plane *S*_0_ (observation region *S*). **b** Illustration of a structurally multiplexed metasurface^30–34,37,38^. The structurally multiplexed metasurface reconstructed several distinguished field distribution ${\mathbf{{U}}}^{({\boldsymbol{n}})}$ based on the metasurface function ${\mathbf{U}}_{{\mathbf{meta}}}^{({\boldsymbol{n}})}$. **c** Schematic illustration of a multi-beam meta-opener^39^. The multi-beam meta-opener is formed by several kinds of “key arrays”, which is used to read out the distributions ${\mathbf{{U}}}^{({\boldsymbol{n}})}$ carried by the preset incident beams. **d** Illustration of transmission-type multi-momentum meta-transformer. The multi-momentum meta-transformer decoder phase profile is implemented with TiO_2_ nano-fin array with in-plane orientations on a quartz substrate. It controls multi-beams with different momenta (*l*^(*n*) ^and $k_0^{\left( n \right)}$) to reconstruct corresponding field distributions ${\mathbf{{U}}}^{({\boldsymbol{n}})}$ Schematic of the OAM meta-transformer. Under the illumination of vortex beams with right circular polarization (RCP), the decoder can generate distinct images at the same plane with left circular polarization (LCP). **f** Schematic of the LM meta-transformer. Under the illumination of different LM beams with RCP, the meta-transformer can generate distinct LM-dependent field distributions at the same region on optical axis. **g** Top-view scanning electron microscopy (SEM) images of a partial region of the fabricated TiO_2_ nano-fins arrays. Scale bar: 2 μm. **h** Oblique-view SEM image. Scale bar: 2 μm. Each TiO_2_ nano-fin represents a phase pixel as defined in the meta-transformer

Multi-tasked metasurface is preferably desired for more compact nanophotonic devices, which could reconstruct multiple distinguished field distributions **U**^(***n***)^ at observed regions. Based on Eq. 1, previously reported multi-tasked metasurfaces can be divided into three categories (shown in Fig. 1a–c). The first approach takes advantage of spatial separation as shown in Fig. 1a. By separating the observed regions (*S*^(*n*)^)^21,22^ or interleaving subarrays $\left( {S_0^{(n)}} \right)$^23–29^ specifically designed for each functionality on the metasurface, the spatial multiplexed metasurface can reconstruct different field distributions (**U**^(***n***)^), but its efficiency is limited. The second approach resorts to adjusting functions ${\mathbf{U}}_{{\mathbf{meta}}}^{\left( {\boldsymbol{n}} \right)}$ of the metasurface by changing polarizations^30–36^, incident angles^37^ or wavelengths^38^ of beams as shown in Fig. 1b. By tailoring meta-atoms, the structurally multiplexed metasurface carries polarization-, angle- or wavelength-dependent responses ${\mathbf{U}}_{{\mathbf{meta}}}^{\left( {\boldsymbol{n}} \right)}$ to reconstruct different field distributions (**U**^(***n***)^). This method can achieve higher efficiency than the previous one, but the efficiency is still limited by the coverage of the required phases that meta-atoms need to achieve. Another approach, as shown in Fig. 1c, is based on OAM multiplexing chip to read out the pre-set incident light (**A**_**inc**_)^39^, which pre-generate various information-carrying multifocal beams arrays with corresponding OAM states. Each ring groove of the chip can out-couple a determined OAM order^40,41^, which equivalently provides a “key” array to read out the distributions **U**^(***n***)^ locked in the preset beams by the spatial light modulator (SLM)^39^. However, the outcoupling efficiency of slits is much smaller than that in the previous two methods and it needs the beam array to illuminate each meta-atom. So far, a majority of reported multi-tasked metasurface are resorting to the spatial freedom, structure complexity or pre-modulation with decoupler, and there is a lack of a convenient way to realize multi-tasked functionality by intrinsic properties of light.

In this paper, we report a transmission-type multi-momentum meta-transformer, which transforms the intrinsic phases of OAM (*lħ*) and LM (*k*_0_*ħ*) of the incidence light, into various distinct patterns in the same plane (Fig. 1d). The meta-transformer is made of the minimalist TiO_2_ nano-fin array (Fig. 1g, h) and provides a phase profile $\psi _{{\mathrm{meta}}}\left( {x_0,y_0} \right) = 2\varphi \left( {x_0,y_0} \right)$ for the right circularly polarized beam with spin angular momentum (SAM). The phase is based on geometric phase and *φ*(*x*_0_, *y*_0_) is the orientation angle of nano-fins as a function of position. For a given fabricated metasurface, incident beams **U**_**inc**_(*x*_0_, *y*_0_) carrying OAM or LM are able to impart extra phase profiles *ψ*_OAM_(*x*_0_, *y*_0_) or affect impulse response *h*(*k*_0_) to the transmitted light to realize distinct patterns in the same plane right on axis. A multi-OAM phase retrieval algorithm is developed to design the OAM meta-transformer, which can “read out” the order of the incident vortex beam. To be more specific, an image which tells the order of the incident vortex beam will be reconstructed at a given plane under illumination of vortex beam with certain orders as shown in Fig. 1e. In addition a multi-LM phase retrieval algorithm is proposed to design a LM meta-transformer, that can reconstruct patterns “R”, “G” and “B” in the same plane by illuminating it with red $\left( {k_0^{\left( {\mathrm{R}} \right)}} \right)$, green $\left( {k_0^{\left( {\mathrm{G}} \right)}} \right)$, and blue $\left( {k_0^{\left( {\mathrm{B}} \right)}} \right)$ beams, respectively (Fig. 1f). This is due to the impulse response in Eq. 1 which is *k*_0_-dependent. Moreover, such LM meta-transformer has demonstrated the capability to display vivid colorful images, thanks to the high-efficiency of the dielectric metasurface and the on-axis imaging feature for all wavelengths.

## Results

### Principle of multi-momentum meta-transformer

**Fig. 2:**
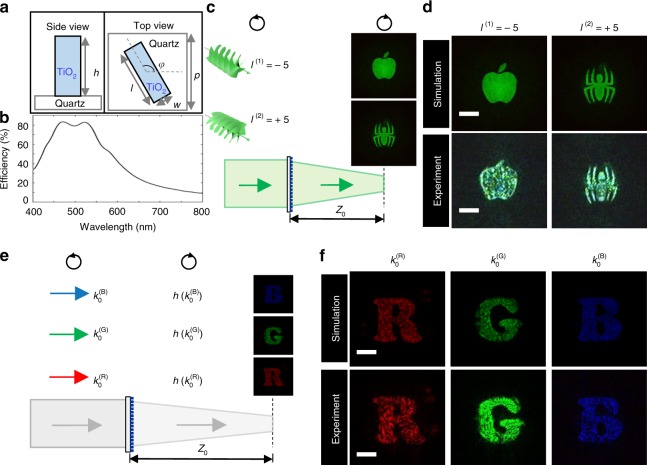
Principle and demonstration of multi-momentum meta-transformer. **a** Geometry of the designed unit cell structure representing one pixel in the meta-transformer, with the periodicity of 325 nm. The TiO_2_ nano-fin parameters are *w* = 80 nm, *l* = 250 nm, and *h* = 600 nm. The in-plane rotating angle *φ* of nano-fin will introduce the geometric phase of 2*φ* for the incident beam with RCP. **b** Measured conversion efficiency of the meta-transformer. The conversion efficiency is defined as the optical power of the transmitted light with opposite CP divided by the incident optical power. **c** Design principle of OAM meta-transformer. Under the illumination of vortex beam 1 (*l*^(1)^ = −5) with RCP, the transmitted beam with opposite CP carries the total phase profile $\psi _{{\mathrm{OAM}} = - 5}\left( {x_0,y_0} \right) + \psi _{{\mathrm{meta}}}\left( {x_0,y_0} \right)$ and reconstructs “apple” pattern in the observation plane. When using vortex beam 2 (*l*^(2)^ = 5) with RCP, the total phase profile of the transmitted beam is $\psi _{{\mathrm{OAM}} = 5}\left( {x_0,y_0} \right) + \psi _{{\mathrm{meta}}}\left( {x_0,y_0} \right)$, which causes the reconstructed pattern change to a spider-shaped pattern. **d** Simulated (top) and measured (bottom) reconstructed patterns by vortex beam 1 (*l*^(1)^ = −5) (left) and vortex beam 2 (*l*^(2)^ = 5) (right). Scale bar: 20 μm. **e** Design principle of LM meta-transformer. Under the illumination of right circularly polarized beam with ${\mathrm{LM}} = k_0^{\left( {\mathrm{R}} \right)}\hbar$, the transmitted beam with opposite CP carry the dispersionless phase profile of metasurface *ψ*_meta_ (*x*_0_, *y*_0_). Due to the convolution of ${\mathbf{U}}_{{\mathbf{inc}}}\left( {x_0,y_0} \right)exp\left( {i\psi _{{\mathrm{meta}}}\left( {x_0,y_0} \right)} \right)$ and impulse response $h\left( {x,y,z,k_0^{\left( {\mathrm{R}} \right)}} \right)$, the transmitted beam reconstructs patterns at the observation plane. Because the impulse response *h *is *k*_0_-dependent, by changing LM of incidence, the reconstructed images “R”, “G” and “B” components can be shifted to one identical plane (*z* = *Z*_0_). **f** Simulated (top) and experimental (bottom) reconstruction of three-primary color holograms at the imaging plane. Scale bar: 20 μm. The original “spider” image was obtained from PNG image website

The metasurface consists of a set of amorphous TiO_2_ nano-fins arranged in square pixels on a quartz substrate (Fig. 2a). The pixel size is 325 × 325 nm^2^ and the TiO_2_ nano-fin is 80-nm-wide, 250-nm-long and 600-nm-high, which rotates in plane with an orientation angle *φ*. When a circularly polarized beam is normally incident on the metasurface from the side of quartz, the transmitted light converts to the opposite circular polarization (CP) and acquires a phase delay of ±2*φ* (Supplementary Note 2). Lumerical FDTD solution is employed to optimize the geometrical parameters of nano-fins, such that the conversion from one CP to the opposite CP is efficient for the operation in broadband visible range (as shown in Fig. 2b).

Figure 2c presents the schematic of OAM meta-transformer. A collimated vortex beam with native phase profile *ψ*_OAM_ (*x*_0_, *y*_0_) illuminates the metasurface with its own as-fabricated phase *ψ*_meta_(*x*_0_, *y*_0_) from the quartz substrate, and the transmitted beam carries the total phase profile $\psi _{\mathrm{T}}\left( {x_0,y_0} \right) = \psi _{{\mathrm{OAM}}}\left( {x_0,y_0} \right) + \psi _{{\mathrm{meta}}}\left( {x_0,y_0} \right)$ on illuminating area (Supplementary Note 3). The metasurface’s phase function *ψ*_meta_(*x*_0_, *y*_0_), which is fixed after the fabrication, is defined by the in-plane orientation of nano-fin array. The extra phase profile *ψ*_OAM_(*x*_0_, *y*_0_) from the incident vortex beam is dynamically changeable. As a degree of freedom of light, by feeding different spiral phases to the single-phase metasurface, one meta-transformer can, therefore, provide multiple different phase profiles, resulting in different patterns on the same plane.

The phase function *ψ*_meta_(*x*_0_, *y*_0_) of OAM meta-transformer is designed by the multi-OAM phase retrieval algorithm (Supplementary Note 4). The orientation angle of each nano-fin is determined by the retrieval phase *ψ*_meta_(*x*_0_, *y*_0_). The schematic diagram of the experimental setup is shown in Supplementary Fig. 3a and its specification can be found in Supplementary Note 6. As shown in Fig. 2d, at the given observation plane (*z* = 60 μm), a judiciously designed metasurface reconstructs an “apple” pattern under illumination with a collimated Laguerre Gaussian beam with *l*^(1)^ = −5, while it shows a “spider” pattern instead when the OAM of the incident beam is changed to *l*^(2)^ = 5. The reconstructed images restore the features of the designed pattern and the crosstalk is suppressed.

Figure 2e presents the physical principle of LM meta-transformer. The phase function *ψ*_meta_(*x*_0_, *y*_0_) of a metasurface is defined by the in-plane orientation of nano-fin array, which means that the designed metasurface is able to provide the dispersionless phase profile. In the Fresnel region, the impulse response *h* of Eq. 1 is

2 $$h\left( {x,y,z} \right) = \frac{{e^{ik_0z}}}{{i\lambda z}}e^{i\frac{{k_0}}{{2z}}\left[ {x^2 + y^2} \right]}$$

Therefore, when collimated monochromatic beams illuminate the metasurface, this dispersionless phase profile *ψ*_meta_(*x*_0_, *y*_0_) can control the monochromatic beams with different LMs to achieve different E-field distribution in a given plane (shown in Fig. 2e).

To realize the LM meta-transformer, the key idea is to reconstruct LM-dependent patterns in the same plane. In the visible region, the monochromatic beams with different LMs represent different colors. So three-primary colors (red 633 nm, green 532 nm, and blue 488 nm) are chosen in this work. Based on the *k*_0_-dependent impulse response, the multi-LM phase retrieval algorithm (Supplementary Note 5) is deployed here to calculate the phase distribution *ψ*_meta_(*x*_0_, *y*_0_). Hence, the wavelength-dependent patterns are reconstructed in the same plane (at *Z*_0_) and the unwanted patterns are moved out of the observed plane. In this design, based on the retrieval phase profile *ψ*_meta_(*x*_0_, *y*_0_), the nano-fins (600 × 600) with rotation angles are fabricated on a total area of 192 × 192 μm^2^. The schematic diagram of the experiment setup of color meta-hologram is shown in Supplementary Fig. 3b and the specification of the experiment setup can be found in Supplementary Note 6. The image is captured by a CCD camera. Figure 2f shows the holographic images of letter patterns “R”,”G”,”B” at a certain distance (*z* =195 μm), corresponding to the RGB color incident beams (633 nm, 532 nm, and 488 nm). The images corresponding to the RGB color incident beams are independent from each other, and the cross-talk among different LMs is eliminated, due to *k*_0_-dependent impulse response.

## Discussion

**Fig. 3:**
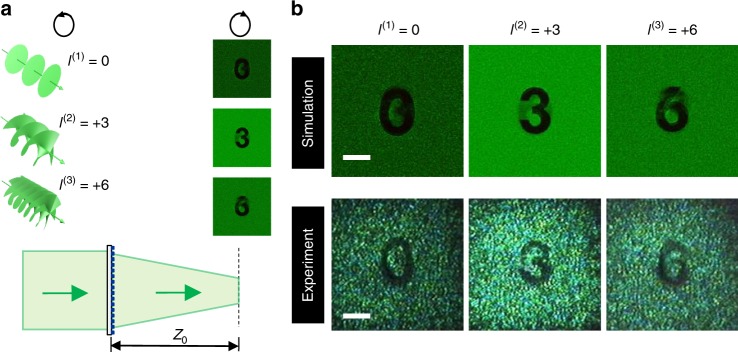
OAM meta-transformer for the read-out of three OAM states. **a** Schematic of OAM meta-transformer designed on three OAM states. This meta-transformer is designed to reconstructed “0”, “3” and “6” patterns at *l*^(*n*)^ = 0, 3, and 6, respectively. **b** Simulated (top) and measured (bottom) reconstructed patterns. Scale bar: 20 μm

The OAM meta-transformer can now work as an OAM displayer, as demonstrated in Fig. 3a. This meta-transformer is formed by 300 × 300 nano-fins on the total area 97.5 × 97.5 μm^2^. As shown in Fig. 3b, the designed OAM meta-transformer reconstructs the patterns “0”, “3” and “6” with the incident 0^th^-, 3^rd^- and 6^th^-order vortex beams, respectively. Compared with Fig. 2d, the OAM meta-transformer in Fig. 3b has lower phase difference among the incident vortex beams and encodes more OAM states, which may reduce the performance. Nevertheless, the reconstructions in Fig. 3b are clearly recognizable. The reconstructed patterns present the incident beam’s topological charge values, which suggests a potential application of OAM topological charge displayer (OAM detection by direct reading) with such OAM meta-transformer.

**Fig. 4:**
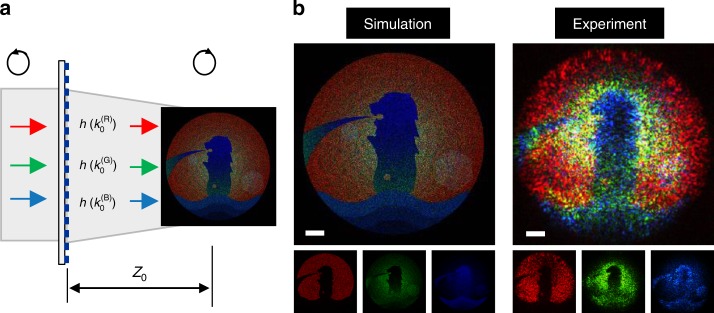
Reconstruction of color images. **a** Schematic illustration of reconstruction of gradient color image. **b** Intensity profiles corresponding to red, green, and blue beams and the graded color holographic image. Scale bar: 30 μm. The original “merlion” image was obtained from Vecteezy.com

Based on *k*_0_-dependent impulse response, the LM meta-transformer generates LM-dependent patterns on the identical position (Fig. 2e, f). Under illumination of red, green and blue beams simultaneously, these generated patterns overlapping each other enable the realization of colorful holographic images (Supplementary Note 7). The colorful holographic images contain not only three-primary colors (RGB) but also their secondary colors (cyan, magenta, yellow, as well as white). Moreover, the LM meta-transformer can also support complex patterns with gradient color (in Fig. 4a, b). Figure 4b reports the simulation and experimental results for each color and their superposition. These results indicate the accurate spatial control of the reconstructed images, and the crosstalk among different LMs is eliminated. The key feature for such gradient color image is that the color gamut spans the whole color triangle. Therefore, the LM meta-transform has the capability to display vivid colorful image.

The capability is a critical issue for the multi-tasked metasurface. Based on the multi-momentum phase retrieval algorithm provided in this work, a single meta-transformer is able to support 5 OAM states ranging from −8 to 8 or 6 LM states in the visible region (Supplementary Note 8). With the help of the polarization-dependent response^21,42,43^ of TiO_2_ nano-fins, the number of patterns encoded in a meta-transformer can increase to 9 for OAM or 11 for LM. Furthermore, the capability of a meta-transformer can also be improved by increasing the number of units^25^, and widening the multi-momentum state region. Besides, by considering the donut shapes of vortex beams, introducing random phase mask^44^, and combining the LM and OAM simultaneously in the phase retrieval algorithm, the multi-momentum meta-transformer has the potential to remarkably increase the carried information states.

In summary, we report a synergetic strategy of engaging OAM and LM of the incident beam with a single-phase metasurface, which significantly enriches the output of reconstructed patterns. OAM introduces the additional phase information from the incident beam, while the LM affects the phase information of impulse response. These two momentum degrees of freedom, in principle lead to infinite combined states, which enables metasurface with a large number of functions and may lead to new opportunities in 3D imaging, anti-counterfeiting, optical communication, and real-time detection.

## Methods

### Numerical simulation

The amorphous TiO_2_ nano-fins were optimized by Lumerical FDTD Solution (a commercial software). In this simulation, TiO_2_ nano-fins with measured refractive index were placed on quartz substrate. Two sources polarized along *x*- and *y*-axes with a 90° phase shift was used to form the right circularly polarized incident beam, and this beam illuminated TiO_2_ nano-fins from the substrate side. The periodic boundary condition was used along *x*- and *y*-directions, and the perfect matched layer (PML) was chosen along *z*-direction.

## Supplementary information

Supplementary Information

Peer Review File

## Data Availability

The data that support the findings of this study are available from the corresponding author upon reasonable request.

## Code availability

The codes that support the plots within this paper and other findings of this study are available from the corresponding authors upon reasonable request.
