# Supplementary material for: Dielectric multi-momentum meta-transformer in the visible
Source: Nat Commun. 2019 Oct 21;10:4789. doi: 10.1038/s41467-019-12637-0 (PMC6803701; doi:10.1038/s41467-019-12637-0)
Supplement: Supplementary file 1 — Supplementary Information [file 41467_2019_12637_MOESM1_ESM.pdf]

SUPPLEMENTARY INFORMATION

Dielectric Multi-Momentum Meta-Transformer in the Visible

Lei Jin[1,⊥], Yao-Wei Huang[2,1,⊥], Zhongwei Jin[1,⊥], Robert C. Devlin[2], Zhaogang Dong[3], Shengtao Mei[1], Menghua Jiang[1], Wei Ting Chen[2], Zhun Wei[1], Hong Liu[3], Jinghua Teng[3], Aaron Danner[1], Xiangping Li[4], Shumin Xiao[5], Shuang Zhang[6], Changyuan Yu[1,7], Joel K.W. Yang[3,8], Federico Capasso[2]*, and Cheng-Wei Qiu[1]*

[1]Department of Electrical and Computer Engineering, National University of Singapore, 4 Engineering Drive 3, 117583, Singapore

[2]Harvard John A. Paulson School of Engineering and Applied Sciences, Harvard University, Cambridge, Massachusetts 02138, United States

[3]Institute of Materials Research and Engineering, A*STAR (Agency for Science, Technology and Research), 2 Fusionopolis Way, #08-03 Innovis, 138634, Singapore

[4]Guangdong Provincial Key Laboratory of Optical Fiber Sensing and Communications, Institute of Photonics Technology, Jinan University, Guangzhou, 510632, People's Republic of China

[5]Ministry of Industry and Information Technology Key Lab of Micro-Nano Optoelectronic Information System, Harbin Institute of Technology, Shenzhen, Guangdong 518055, People's Republic of China

[6]School of Physics and Astronomy, University of Birmingham, Birmingham B15 2TT, UK

[7]Department of Electronic and Information Engineering, The Hong Kong Polytechnic University, Hung Hom, Kowloon, Hong Kong

[8]Singapore University of Technology and Design, 8 Somapah Road, 487372 Singapore

Corresponding authors: C.-W. Qiu (email: chengwei.qiu@nus.edu.sg) and Federico Capasso (email:capasso@seas.harvard.edu).

Supplementary Note 1. The diffraction field for vector beams

In this work, (x_0, y_0) and (x, y) are the spatial coordinates at the metasurface and observed planes, respectively. z is distance between the metasurface and observed region. A polarized light $\mathbf{U_{inc}}(x_0, y_0)$ can be expressed as Jones vector $\left[E_{x_0}(x_0, y_0), E_{y_0}(x_0, y_0)\right]'$, where E_{x_0}, E_{y_0} are complex numbers and " $'$ " denotes matrix transposition. For a metasurface, its transmission/reflection function $\mathbf{U_{meta}^{T/R}}(x_0, y_0)$ can be expressed as Jones matrix:

$$\mathbf{U_{meta}^{T/R}}(x_0, y_0) = \begin{bmatrix} u_{x_0x_0}^{\mathrm{T/R}}(x_0, y_0) & u_{x_0y_0}^{\mathrm{T/R}}(x_0, y_0) \\ u_{y_0x_0}^{\mathrm{T/R}}(x_0, y_0) & u_{y_0y_0}^{\mathrm{T/R}}(x_0, y_0) \end{bmatrix}, \tag{1}$$

where $u_{mn}^{\mathrm{T/R}}$(m, n denotes x_0, y_0) is complex number. When the polarized monochromatic light $\mathbf{U_{inc}}(x_0, y_0)$ illuminate the metasurface $\mathbf{U_{meta}^{T/R}}(x_0, y_0)$, according to Eq. 1 in the main text, the field in the observation plane is

$$\mathbf{U}(x, y, z) = \iint_{-\infty}^{+\infty} \mathbf{U_{meta}^{T/R}}(x_0, y_0)\mathbf{U_{inc}}(x_0, y_0)h(x - x_0, y - y_0, z)dx_0dy_0, \tag{2}$$

where

$$\mathbf{U_{meta}^{T/R}}(x_0, y_0)\mathbf{U_{inc}}(x_0, y_0) = \begin{bmatrix} u_{x_0x_0}^{\mathrm{T/R}}(x_0, y_0) & u_{x_0y_0}^{\mathrm{T/R}}(x_0, y_0) \\ u_{y_0x_0}^{\mathrm{T/R}}(x_0, y_0) & u_{y_0y_0}^{\mathrm{T/R}}(x_0, y_0) \end{bmatrix} \begin{bmatrix} E_{x_0}(x_0, y_0) \\ E_{y_0}(x_0, y_0) \end{bmatrix}. \tag{3}$$

When the incident light is circular polarized and carries OAM ($l\hbar$), the Jones vector of incident light adds a new variable l and can be expressed as

$$\mathbf{U_{inc}}(x_0, y_0, l) = \mathbf{A_{inc}}(x_0, y_0)e^{il\theta(x_0, y_0)} = \begin{bmatrix} 1 \\ \pm i \end{bmatrix} A(x_0, y_0)e^{il\theta(x_0, y_0)}, \tag{4}$$

where, $\mathbf{A_{inc}}(x_0, y_0)$ denotes the intensity profile $A(x_0, y_0)$ with polarization state, and $\theta(x_0, y_0)$ indicates azimuthal angle. For the incident light with LM ($k_0\hbar$), the impulse

response h changes with k_0. According to the k_0-dependent impulse response $h(x, y, z, k_0)$, and substituting Eq. (4) into Eq. (2), we can obtain:

$$\mathbf{U}(x, y, z, l, k_0) = \iint_{-\infty}^{+\infty} \mathbf{U}_{\mathbf{meta}}^{\mathbf{T/R}}(x_0, y_0)\mathbf{A}_{\mathbf{inc}} e^{il\theta(x_0,y_0)} h(x - x_0, y - y_0, z, k_0) dx_0 dy_0, \quad (5)$$

where

$$\mathbf{U}_{\mathbf{meta}}^{\mathbf{T/R}}(x_0, y_0)\mathbf{A}_{\mathbf{inc}} e^{il\theta(x_0,y_0)} = \begin{bmatrix} u_{x_0x_0}^{\mathrm{T/R}}(x_0, y_0) & u_{x_0y_0}^{\mathrm{T/R}}(x_0, y_0) \\ u_{y_0x_0}^{\mathrm{T/R}}(x_0, y_0) & u_{y_0y_0}^{\mathrm{T/R}}(x_0, y_0) \end{bmatrix} \begin{bmatrix} 1 \\ \pm i \end{bmatrix} A(x_0, y_0) e^{il\theta(x_0,y_0)}. \quad (6)$$

Supplementary Note 2. Geometric phase introduced by the nano-fin

In this work, the designed metasurfaces are transmission type and formed by single-sized TiO_2 nano-fin array. The single TiO_2 nano-fin is designed as a half-wave plate (HWP) and has in-plane orientation angle φ. Then, the Jones matrix for this nano-fin can be expressed as

$$\mathbf{U}_{\mathbf{nano-fin}}^{\mathbf{T}}(\varphi) = \mathbf{R}(-\varphi)\mathbf{T_0}\mathbf{R}(\varphi) = \begin{bmatrix} cos^2\varphi - sin^2\varphi & 2cos\varphi sin\varphi \\ 2cos\varphi sin\varphi & sin^2\varphi - cos^2\varphi \end{bmatrix}, \quad (7)$$

where $\mathbf{T_0}$ is the Jones matrix of HWP and $\mathbf{R}(\varphi)$ is the rotation operator. From Eq. (7), the transmission function of designed metasurface can be written as:

$$\mathbf{U}_{\mathbf{meta}}^{\mathbf{T}}(x_0, y_0) = \begin{bmatrix} u_{x_0x_0}^{\mathrm{T}}(x_0, y_0) & u_{x_0y_0}^{\mathrm{T}}(x_0, y_0) \\ u_{y_0x_0}^{\mathrm{T}}(x_0, y_0) & u_{y_0y_0}^{\mathrm{T}}(x_0, y_0) \end{bmatrix}$$

$$= \begin{bmatrix} cos^2\varphi(x_0, y_0) - sin^2\varphi(x_0, y_0) & 2cos\varphi(x_0, y_0)sin\varphi(x_0, y_0) \\ 2cos\varphi(x_0, y_0)sin\varphi(x_0, y_0) & sin^2\varphi(x_0, y_0) - cos^2\varphi(x_0, y_0) \end{bmatrix} \quad (8)$$

Then, we can deduce the expression:

$$\begin{bmatrix} u_{x_0x_0}^{\mathrm{T}}(x_0, y_0) & u_{x_0y_0}^{\mathrm{T}}(x_0, y_0) \\ u_{y_0x_0}^{\mathrm{T}}(x_0, y_0) & u_{y_0y_0}^{T}(x_0, y_0) \end{bmatrix} \begin{bmatrix} 1 \\ \pm i \end{bmatrix} = e^{i2(\pm\varphi(x_0,y_0))} \begin{bmatrix} 1 \\ \mp i \end{bmatrix}, \quad (9)$$

Therefore, a TiO_2 nano-fin with a rotating angle will introduce the geometric phase $\pm 2\varphi(x_0, y_0)$ for opposite spins. Substituting Eq. (9) into Eq. (5), we can obtain:

$$\mathbf{U}(x, y, z, l, k_0) = \iint_{-\infty}^{+\infty} e^{i2(\pm\varphi(x_0,y_0))} \begin{bmatrix} 1 \\ \mp i \end{bmatrix} A(x_0, y_0) e^{il\theta(x_0,y_0)} h(x - x_0, y - y_0, z, k_0) dx_0 dy_0 \tag{10}$$

Supplementary Note 3. Expression of the electric field distribution after multi-OAM meta-transformer

The multi-OAM meta-transformer is designed within Fresnel range. Based the Fresnel diffraction approximation, the electric field distribution $\mathbf{U}(x, y, z, l)$ after the multi-OAM meta-transformer can be calculated by:

$$\mathbf{U}(x, y, z, l) = \frac{e^{ikz}}{i\lambda z} \iint \mathbf{U}_{\mathbf{meta}}^{\mathbf{T}}(x_0, y_0) \mathbf{U}_{\mathbf{0_{OAM}}}(x_0, y_0, l) e^{i\frac{k}{2z}[(x-x_0)^2+(y-y_0)^2]} dx_0 dy_0 \tag{11}$$

$$\mathbf{U}_{\mathbf{meta}}^{\mathbf{T}}(x_0, y_0) \mathbf{U}_{\mathbf{0_{OAM}}}(x_0, y_0, l) \propto r^{|l|} \mathrm{e}^{-\frac{r^2}{w^2}} e^{i(\psi_{\mathrm{meta}}+\psi_{\mathrm{OAM}})} \begin{bmatrix} 1 \\ \mp i \end{bmatrix} \tag{12}$$

where $\mathbf{U}_{\mathbf{0_{OAM}}}(x_0, y_0, l)$ is the incident electric field determined by the illuminating OAM beam, and $\mathbf{U}_{\mathbf{meta}}^{\mathbf{T}}(x_0, y_0)$is transmission function of the designed metasurface.

Supplementary Note 4. Multi-OAM phase retrieval algorithm and image reconstruction.

Supplementary Figure 1 shows the simulation of OAM meta-transformer. Supplementary Figure 1 (a) presents the multi-OAM Gerchberg-Saxton algorithm[1]. For the case of several OAM states used in design, this algorithm consists of FT forward propagation with different OAM spiral phase, replacement in the “imaging plane”, IFT backword propagations, and

normalization in the "metasurface plane". In this algorithm, a hybrid strategy[2] is employed at the replacement in the "imaging plane". In the first state (Replacement 1), the calculated amplitude of "imaging plane" $A_{\text{calculated}}(x,y)$ is replaced by the target amplitude distribution $A_{\text{target}}(x,y)$. In the second state (Replacement 2), $[2\delta \times A_{\text{target}}(x,y) - A_{\text{calculated}}(x,y)]$ is used to replace $A_{\text{calculated}}(x,y)$, where factor δ is a ratio between summations of $A_{\text{calculated}}(x,y)$ and $A_{\text{target}}(x,y)$. After enough iterations, the phase profile on metasurface will converge finally. Then, OAM spiral phases are employed to verify the retrieved phase on metasurface, as shown in Supplementary Figure 1 (b).

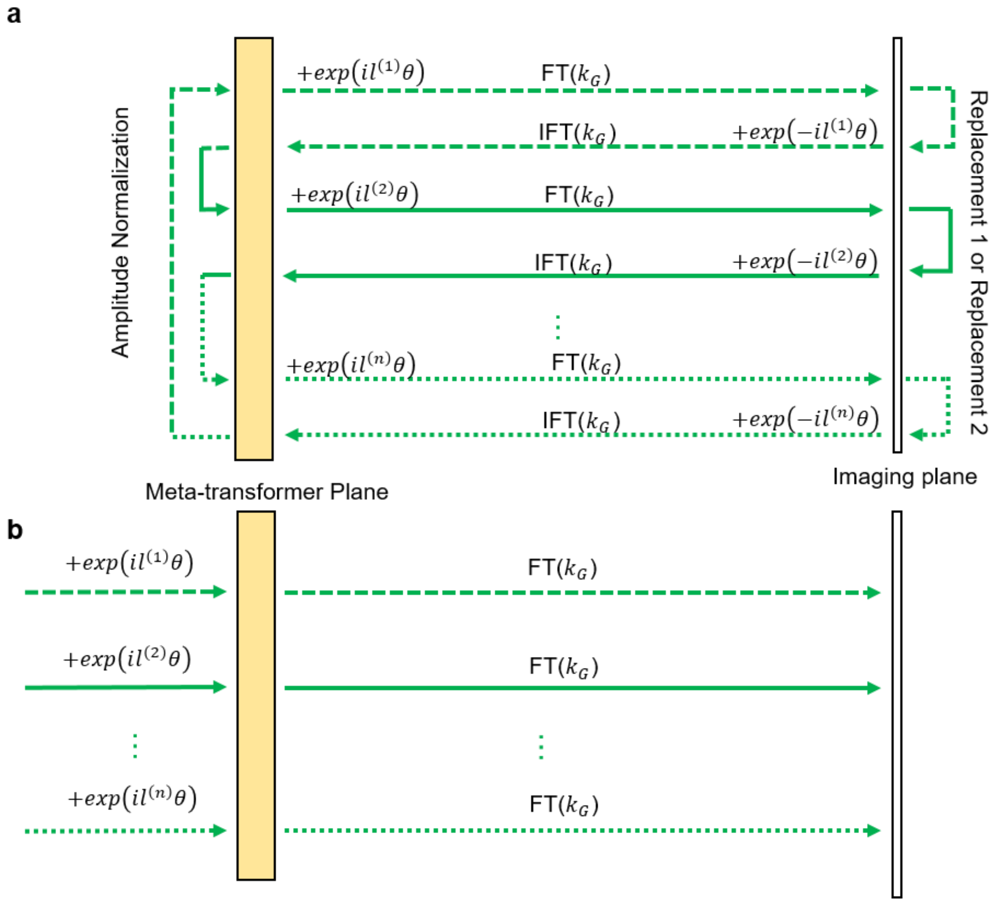

Supplementary Figure 1| Multi-OAM phase retrieval algorithm and image reconstruction. (a) Flow chart of the multi-OAM Gerchberg-Saxton algorithm. This loop is formed by n steps

labelled by square dot, solid and round dot lines, which represent the phase retrieval for beams with OAM = $l^{(1)}$, $l^{(2)}$ and $l^{(n)}$, respectively. FT: Fresnel transformation, IFT: inverse Fresnel transformation. (b) Images verification process.

Supplementary Note 5. Multi-LM phase retrieval algorithm and image reconstruction

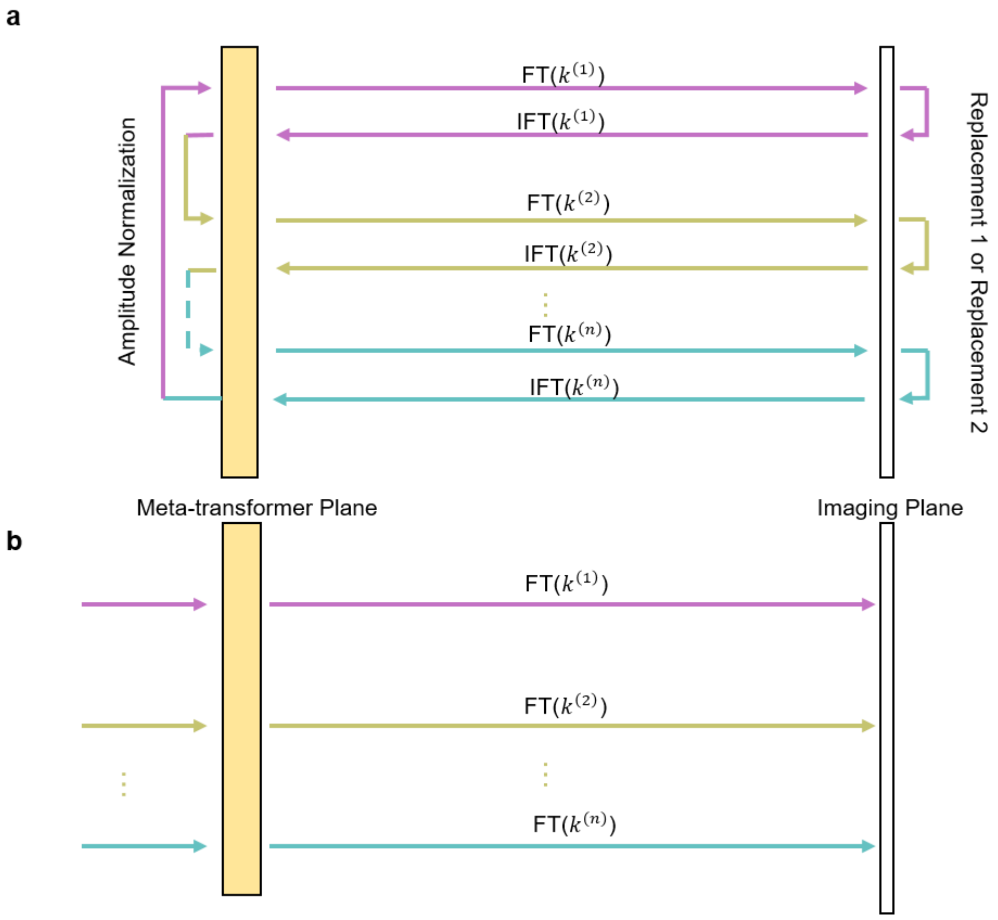

Supplementary Figure 2 | Multi-LM phase retrieval algorithm and image reconstruction. (a) Flow chart of the multi-LM Gerchberg-Saxton algorithm. This loop is formed by *n* steps labelled by different colors, which represent the phase retrieval for different LMs. FT: Fresnel transformation, IFT: inverse Fresnel transformation. (b) Image verification process.

As shown in Supplementary Figure 2 (a), this multi-LM Gerchberg-Saxton algorithm[1] is a serial iterative algorithm. For the case of *n* LM states used in design, every loop is formed by *n* steps. Every step uses the FT and iFT for the given LM, does replacement at “image plane” and normalizes amplitude at “metasurface plane” each time. The replacement at “image plane” is same to the multi-OAM Gerchberg-Saxton algorithm in Supplementary Note 4. After enough iterations, the optimized phase profile can be achieved to reconstruct multi-LM images at the given plane. Supplementary Figure 2 (b) shows multi-LM beams are employed to verify the retrieved phase on metasurface.

Supplementary Note 6. Experiment Setup

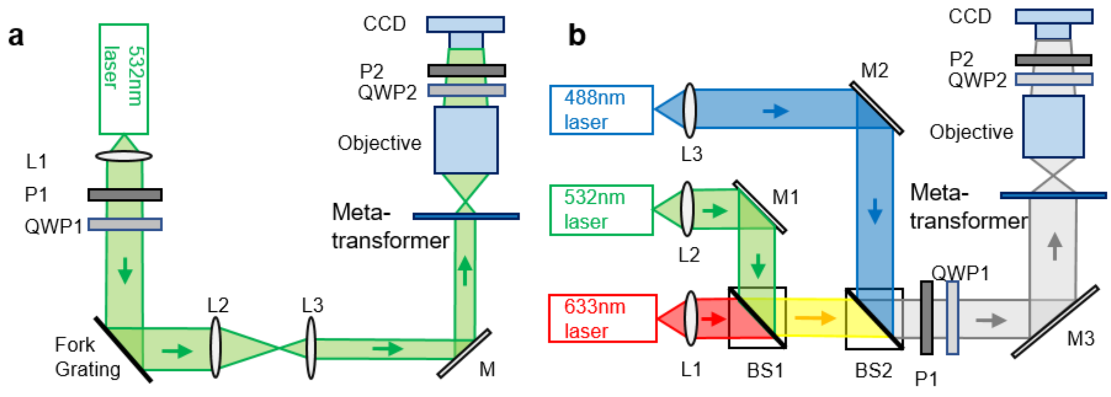

Supplementary Figure 3 | Experimental setup. (a) Experimental setup for the OAM readable meta-hologram. Fork grating is used to modify the incident beam with OAM. (b) Experimental setup for the full-color meta-hologram. Two beam splitters are used to combine three primarily colour beams. The polarization states of the incident beams are tuned by controlling the angle between the polarization axis of a polarizer (P1) and the fast axis of the quarter-wave plate (QWP1). The reconstructed holographic images are encoded in the transmitted cross-polarized beams, which are collected by objective lens with a set of QWP 2 and P2 before the CCD.

Supplementary Figure 3 (a) shows the experiment setup for the OAM meta-transformer. A laser emitting at 532 nm is used in this experiment. After being collimated by lenses, the laser beam travel through a circular polarizer to generate circularly polarized beam. The circular polarizer consists of a broadband linear polarization and a quarter-wave plate (QWP) worked at 532 nm. The fork grating, fork-shaped binary computer-generated hologram[3], is employed here to generate vortex beams. The vortex beam is actually normally incident from the bottom side after resized by two lenses in order to have similar size to the metasurface sample. The reconstructed images are formed by transmitted spin-inversed beams. The transmitted spin-inversed lights are collected by an objective lens, then filtered by another circular-polarization analyser, and final captured by a CCD camera.

The schematic diagram of the experiment setup for the LM meta-transformer is shown in Supplementary Figure 3 (b). Three lasers (emitting at 488 nm, 532 nm and 633 nm) are collimated by lenses (L1, L2 and L3). After reflected by mirrors (M1 and M2), these three laser beams are combined together by two beam splitters (BS1 and BS2). The combined beams pass through a broadband linear polarization (P1) and quarter-wave plate (QWP1) to achieve circular polarization. The circularly polarized beams illuminate LM meta-transformer from substrate side. The transmitted beams with opposite spin reconstruct the images and those images captured by a CCD camera.

Supplementary Note 7. Full color images

Supplementary Figure 4 (a) shows the schematic illustration of reconstruction of full-color meta-hologram. The fabricated metasurface is also formed by the nano-fins (600×600) arranged on a total area of 192×192 μm^2. Under the illumination of red, green and blue beams,

the metasurface can generate a full-color meta-hologram in the observed plane. The reconstructed intensity profiles corresponding to red, green and blue beams and the full-color holographic image are displayed in Supplementary Fig. 4 (b). The reconstructed image contains not only three-primary color (RGB) but also their secondary colors. The secondary colors, including the cyan, magenta and yellow colors, as well as the white color where all three primary colors overlap with each other on axis, have been successfully reproduced. These experimental results indicate the accurate spatial control of the reconstructed images.

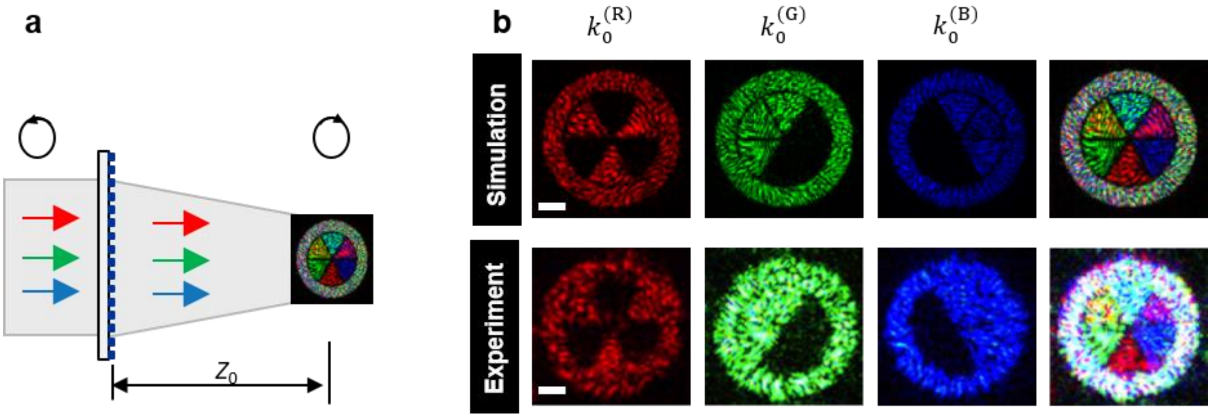

Supplementary Figure 4| Reconstruction of color images. (a) Schematic illustration of reconstruction of full-color image. Under the illumination of red, green and blue beams, the metasurface can generate a full-color image in the imaging plane. (b) Intensity profiles corresponding to red, green and blue beams and the full-color holographic image. Scale bar: 5 µm.

Supplementary Note 8. The capability of multi-momentum meta-transformer

In this simulation, the number of units in a phase profile is fixed at 600×600.

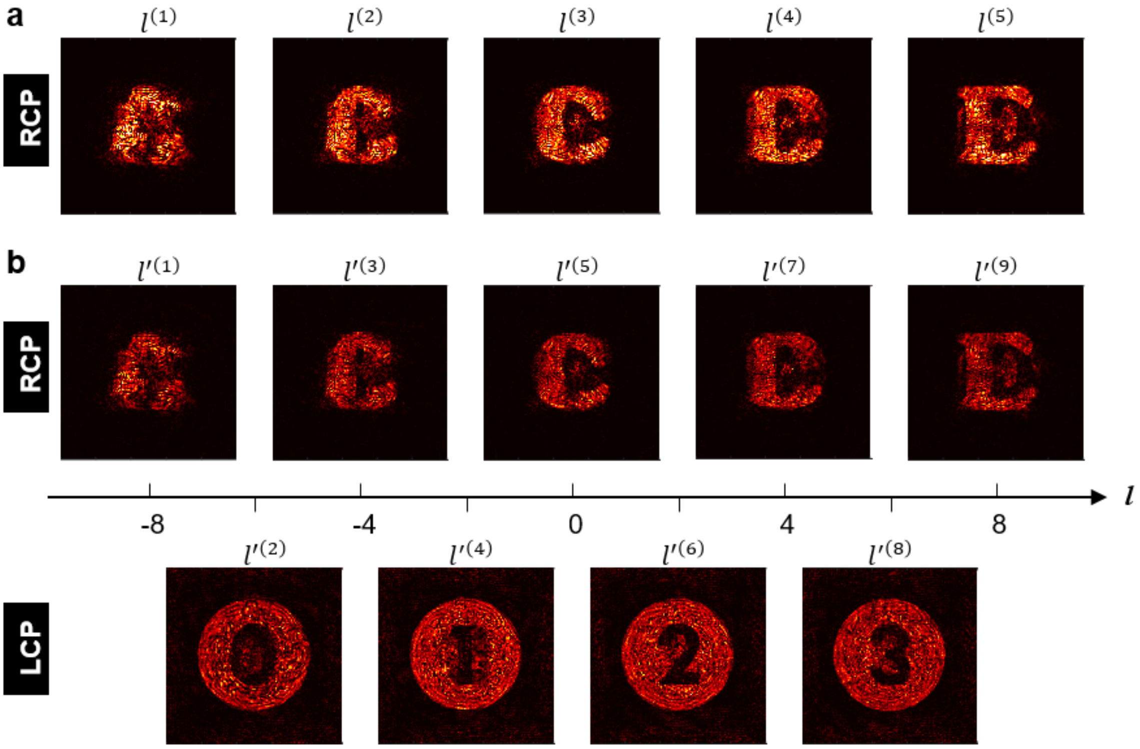

Supplementary Figure 5| The capability of an OAM meta-transformer. (a) The OAM meta-transformer designed for the beam with RCP. This meta-transformer encodes 5 states and $l^{(n)}$ are from -8 to 8 with step 4. (**b**) The OAM meta-transformer designed for the beam with CP. With the help of polarization, a single OAM meta-transformer is able to reconstruct 9 patterns with $l^{(n)}$ ranging from -8 to 8.

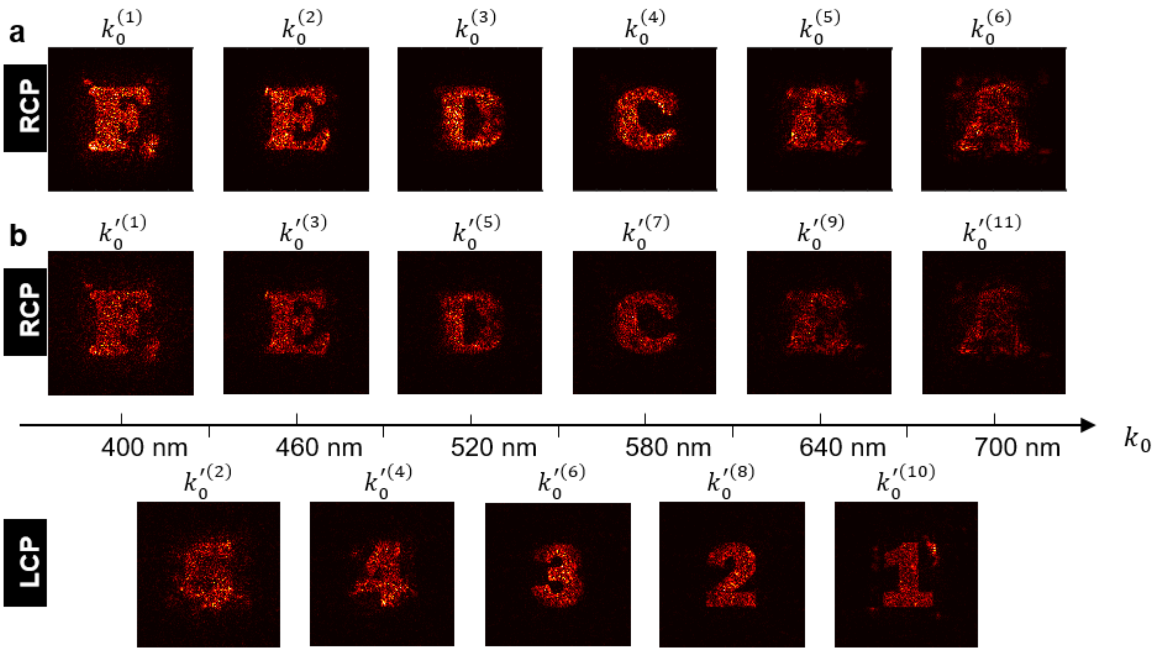

Supplementary Figure 6| The capability of a LM meta-transformer. (a) The LM meta-transformer designed for the beam with RCP. In the visible region, a single LM meta-transformer is capable of supporting 6 LM sates. **(b)** The LM meta-transformer designed for the beam with CP. The capability of a single LM meta-transformer can be increased to 11 LM states.

Supplementary References

1 Gerchberg, R. W. A practical algorithm for the determination of phase from image and diffraction plane pictures. *Optik* **35**, 237-246 (1972).

2 Chen, W. & Chen, X. Optical multiple-image encryption based on multiplane phase retrieval and interference. *Journal of Optics* **13**, 115401 (2011).

3 Stoyanov, L., Topuzoski, S., Stefanov, I., Janicijevic, L. & Dreischuh, A. Far field diffraction of an optical vortex beam by a fork-shaped grating. *Optics Communications* **350**, 301-308 (2015).
